# Is dialdehyde starch a valuable cross-linking agent for collagen/elastin based materials?

**DOI:** 10.1007/s10856-016-5677-6

**Published:** 2016-02-17

**Authors:** J. Skopinska-Wisniewska, K. Wegrzynowska-Drzymalska, A. Bajek, M. Maj, A. Sionkowska

**Affiliations:** Faculty of Chemistry, Nicolaus Copernicus University in Torun, Gagarina 7, 87-100 Toruń, Poland; Collegium Medicum, Nicolaus Copernicus University in Torun, Karlowicza 24, 85-092 Bydgoszcz, Poland

## Abstract

Collagen and elastin are the main structural proteins in mammal bodies. They provide mechanical support, strength, and elasticity to various organs and tissues, e.g. skin, tendons, arteries, and bones. They are readily available, biodegradable, biocompatible and they stimulate cell growth. The physicochemical properties of collagen and elastin-based materials can be modified by cross-linking. Glutaraldehyde is one of the most efficient cross-linking agents. However, the unreacted molecules can be released from the material and cause cytotoxic reactions. Thus, the aim of our work was to investigate the influence of a safer, macromolecular cross-linking agent—dialdehyde starch (DAS). The properties of hydrogels based on collagen/elastin mixtures (95/5, 90/10) containing 5 and 10 % of DAS and neutralized via dialysis against deionized water were tested. The homogenous, transparent, stiff hydrogels were obtained. The DAS addition causes the formation of intermolecular cross-linking bonds but does not affect the secondary structure of the proteins. As a result, the thermal stability, mechanical strength, and, surprisingly, swelling ability increased. At the same time, the surface properties test and in vitro study show that the materials are attractive for 3T3 cells. Moreover, the materials containing 10 % of DAS are more resistant to enzymatic degradation.

## Introduction

Structural proteins, such as collagen and elastin, have been in the focus of research for many years. They possess many excellent properties which can be employed in biomaterials engineering. Collagen in its native state, due to its triple helical structure, is mechanically strong whereas elastin exhibits unusual elasticity [1–6]. Both of them are biocompatible, biodegradable, and promote cells adhesion. Moreover, collagen exhibits low cross species immunogenicity [1, 7, 8]. They exist together in the extracellular matrix (ECM) in the majority of connective tissues and may be used in biomaterials field as a decellularized scaffold [1]. However, pure proteins are used more often. Unfortunately, the isolation of collagen and elastin affects their properties due to the destruction of the ordered structures. Very often, the materials require cross-linking in order to improve their mechanical strength and degradation rate.

**Fig. 1:**
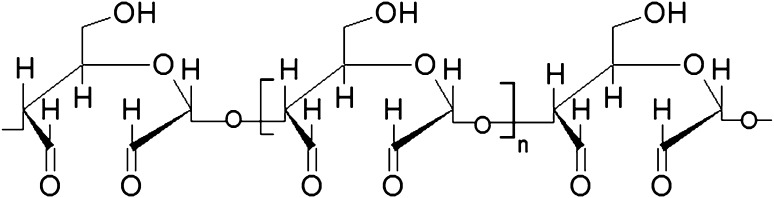
Dialdehyde starch structure

Protein materials may by modified by physical and chemical methods, e.g. UV-light irradiation, dehydrothermal treatment [9–12], the use of natural and synthetic cross-linking agents [7, 8, 13] or enzymes [14]. The chemical cross-linking techniques are considered to be most effective and predictable. Commonly used reagents include carbodiimides, isocyanates, tannic acid, divinylsulphone, diglycidylether, etc. [7, 9, 15–18]. Dialdehydes are another class of valuable crosslinkers. They easily react with amine groups of proteins in mild conditions, at physiological pH, with high efficiency and do not alter the triple helical structure and biological function of collagen molecules. For many years, glutaraldehyde was probably the most widely used cross-linking agent in the field of biomaterials [1, 15, 19, 20]. However, the unreacted dialdehyde molecules which can stay in the scaffold are cytotoxic and may cause undesirable reactions in the patient’s body [21, 22]. Macromolecular dialdehydes are considered as a safer cross-linking agent. Dialdehyde starch (DAS) is obtained due to selective periodate oxidation of starch. During the reaction, the C2–C3 bonds of the glucose units are cleaved and two aldehyde groups are formed (Fig. 1). DAS is biodegradable and toxically acceptable. Moreover, it demonstrates antiviral activity [22–26].

In the present study, we have prepared collagen/elastin hydrogel through dialysis against deionized water and cross-linked by DAS. The gel structure and properties have been tested.

## Materials and methods

### Materials preparation

Collagen was isolated from the tail tendons of young albino rats according to the method previously employed [4]. The tendons were mechanically separated from adhering tissues, washed in deionized water and placed in the 0.1 M acetic acid for 72 h at 8 °C. The insoluble parts were separated by centrifugation at 10000 rpm in an Eppendorf Centrifuge. The collagen solution was then freeze-dried.

Elastin from porcine aorta was purified by Lansing’s method as described [27]. Pig aortas, provided by a local butcher, were cleaned from adhering tissues and cut into small pieces (approx. 0.5 cm wide rings). The whole remaining fat was removed by sequential extractions in ethanol (twice), a mixture of ethanol/ether (50/50) (twice) and ether (also twice). The 50 g of de-fatted tissue was placed in 100 ml of 0.1 M NaOH and heated to 95 °C for 50 min in a water bath under continuous stirring. After cooling at room temperature, the samples were rinsed twice with cold 0.1 M NaOH in a Buchner funnel and then with deionised water. The dry material was minced in liquid nitrogen. The elastin powder (1 g) was suspended in a mixture of 50 ml tert-butanol and 50 ml 1 M KOH, and was stirred for 48 h at room temperature. Next, 50 ml of water was added and the resulting solution was neutralized with acetic acid. The solution of elastin hydrolysates was then dialyzed against deionised water and then lyophilized.

The DAS was supplied from Chemos GmbH, Germany.

### Hydrogel preparation

1 % collagen solution in 0.1 M acetic acid and 1 % elastin hydrolysates solution in water were prepared and mixed in appropriate volume ratios (95/5, 90/10). Then, 5 or 10 % (weight percent based on the dry weight of the protein) of the DAS was added and incubated for 30 min in a magnetic stirrer. Next, the blends were dialysed (Servapor dialysis tube, MWCO 12,000–14,000RC) against deionized water for 7 days (until the pH of the surrounding solution did not change). At the same time, spontaneous hydrogel formation via neutralization process and additional stabilization caused by the cross-linking with the use of DAS took place [28].

The names of all the obtained hydrogels are coded as shown: collagen content—elastin content—DAS addition, e.g. 90Coll-10El-5DAS.

### Infrared spectroscopy

The thin slides of collagen/elastin gels were dried in air. The FTIR-ATR spectra of samples were obtained with the Mattson Genesis II spectrophotometer (USA) equipped with an ATR tool with the 4 cm^−1^ resolution. The FTIR spectra were compared using the program provided by the manufacturer.

### Thermal analysis

The thermal properties of unmodified and cross-linked collagen/elastin materials were studied using a differential scanning calorimeter (DSC 204 F1 Phoenix, Netzsch, Germany). Standard aluminium DSC pans were used with about 1.5 mg samples of the dried material. All the samples were held at 20 °C for 3 min and then scanned at the temperature range from 20 to 250 °C and at the heating rate of 10 °C/min in the atmosphere of nitrogen.

### SEM images

The morphology images of lyophilized collagen/elastin biomaterials were obtained with the use of a scanning electron microscope manufactured by LEO Electron Microscopy Ltd, England, 1430 VP model. The cylindrical samples of fresh hydrogel were cut into slices about 0.5 cm thick and frozen at −20 °C. Then the material was lyophilized under following conditions: main drying phase—pressure 5.1 mBa, temp. of ice condenser −2 °C, time 24 h; final drying phase—pressure 0.01 mBa, temp. of ice condenser −60 °C, time 2 h. The small piece (3 mm × 3 mm) of the material was cut from the middle of the sample. The cross-section perpendicular to long axis of the cylindrical hydrogel was observed. All the samples were coated with gold.

The pore size was analysed using SigmaScan Pro programme. The value was an average at least of five measurements.

### Mechanical properties

Mechanical tests were carried out on fresh hydrogel samples cut into pieces about 1 cm thick. The mechanical properties were determined using a Zwick & Roell Z 0.5 machine (Germany). The collagen/elastin gel samples were placed on the bottom disk and pushed by a steel rod (5 mm diameter) at the speed of 10 mm/min, until 20 % deformation of the material.

### Swelling ability

The swelling ability (E_s_) was measured by the conventional gravimetric method. The dry sample was weighed and placed in 4 ml of 0.05 M phosphate buffer saline (PBS) at pH 7.4, at room temperature. After the appropriate incubation time (1, 2, 4, 5, 6, 24, 48, 72 h), the excess of the phosphate buffer was removed with the use of absorbent paper and the wet material was weighed (W_s_). The liquid content of the scaffolds was defined as the ratio of weight increase (W_s_ − W_d_) with respect to the initial weight (W_d_) of dry samples. Each value was averaged from three parallel measurements. E_s_ was calculated with the following equation:

$${\text{E}}_{\text{s}} = \, \left( {{\text{W}}_{\text{s}} {-}{\text{ W}}_{\text{d}} } \right)/{\text{W}}_{\text{d}}$$

where W_s_ and W_d_ denote the weights of swollen and dry samples, respectively.

### Contact angle and surface free energy

The contact angle of the two liquids, glycerol (G) and diiodomethane (D), on the collagen/elastin surface were measured at constant temperature (22 °C) using a goniometer equipped with the system of drop-shape analysis (DSA produced by Kruss, Germany). Each contact angle is the average value obtained from at least 10 measurements. The surface free energy (SFE) was calculated with the Owens–Wendt method which is one of the most commonly used calculation methods for polymeric materials. The method is based on the assumption that SFE includes two components—a polar ($\gamma_{\text{s}}^{\text{p}}$) and a dispersive ($\gamma_{\text{s}}^{\text{d}}$) component. The contact angle has to be tested with the use of two measuring liquids of different (polar and non-polar) nature. Glycerol (G) and diiodomethane (D) are a widely used pair of liquids for the water swollen materials testing [29].

### Enzymatic degradation

The lyophilized samples of the collagen/elastin material were weighed (W_b_). Then, they were immersed in 1 ml of 0.1 M Tris–HCl buffer (pH 7.4) containing 50 mM CaCl_2_, and left at the temperature of 37 °C for 0.5 h. Next, 1 ml of 0.1 M Tris–HCl buffer (pH 7.4) containing 50 U of bacterial enzyme–collagenase from *Clostridium histolyticum* (Sigma-Aldrich) was added.

After 1 h incubation at 37 °C the samples were placed in 1 ml of 0.25 M EDTA, cooled in ice, rinsed 3 times with deionized water, frozen, lyophilized, and weighed (W_a_).

The degradation degree was calculated from the equation:

$${\text{Degradation degree }}\left( \% \right) \, = \, \left( {{\text{W}}_{\text{b}} - {\text{W}}_{\text{a}} } \right)/{\text{W}}_{\text{b}} *100.$$

The value was an average of three measurements.

### In vitro analysis

In order to investigate the viability and proliferation of 3T3 cells on collagen/elastin matrices, the hydrogels slides were placed in 12-well plates and then the cells were seeded on the top of the material at the density of 2.5 × 10^4^ per well. Cells were growing in a complete medium for 7 days. After that, the culture medium was removed and the MTT assay was performed.

### Statistical analysis

In paper ANOVA one-way test was used with Bonferroni’s post-test. All the data were presented as mean ± SD. Differences were considered significant when the *P* value was <0.05. The confidence interval was 95 %.

## Results

### FTIR spectroscopy

**Fig. 2:**
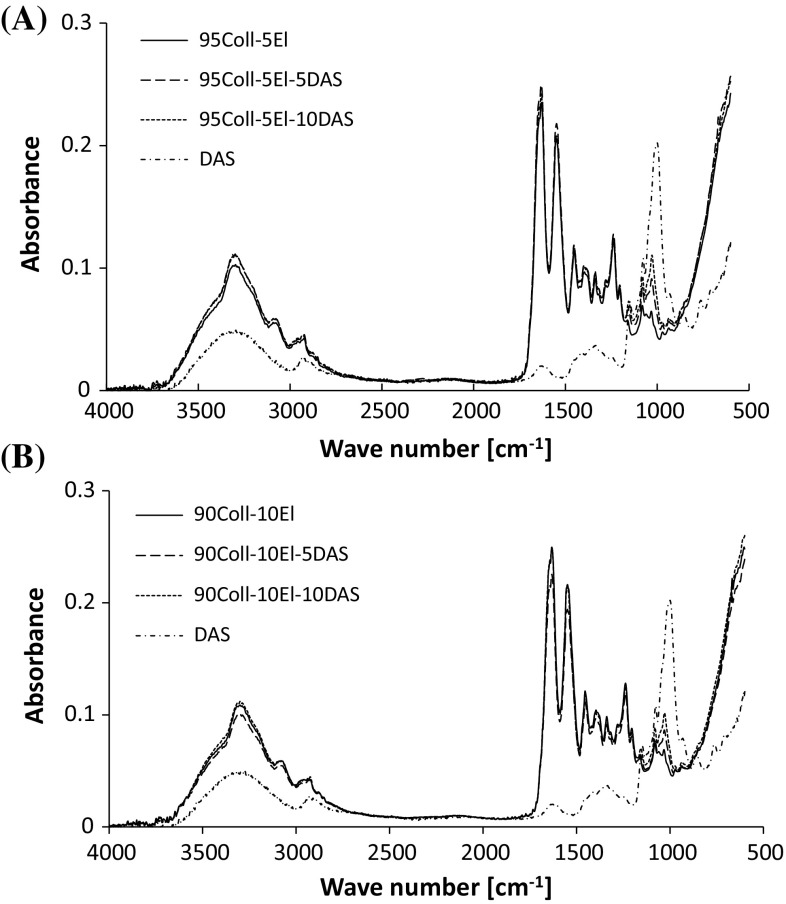
The FTIR spectra of **a** 95Coll-5El, **b** 90Coll-10El materials and DAS

Infrared spectroscopy is a valuable tool to study proteins. The analysis of the position and shape of characteristic bands provides information about molecular structures and conformational changes which follow the cross-linking process. The FTIR spectra of collagen/elastin materials are presented in Fig. 2. The observed bands are typical of proteins. The NH– stretching vibrations rise to the Amide A band at 3298 cm^−1^. This is a part of Fermi resonance with Amide B band (3083 cm^−1^) which represents the overtone of Amide II vibration. The NH– vibrations frequency depends on the strength of the hydrogen bonds which stabilize the protein structure. A very small shift of Amide B position to the lower wave number is noticed for the cross-linked collagen/elastin materials (Table 1). Therefore, the slight, partial reorganization of the hydrogen bonds network is assumed. Amide II (1549 cm^−1^) and Amide III (1238 cm^−1^) may give some information about the polypeptide conformation. However, Amide I is the most sensitive indicator of the secondary structure. It arises from the stretching vibration of C=O (80 %), N–H (20 %), and N–H bending vibrations (10 %) of the peptide bonds [30–33]. Its position at 1631 cm^−1^ observed in collagen/elastin spectra is typical of the triple helical structure of the fresh rat tail tendon [28, 34]. This position and the shape of the band are not significantly changed after collagen/elastin materials cross-linking with DAS (Table 1; Fig. 2). However, the shift of C–O stretching vibrations of DAS from 1002 to 1032 cm^−1^ in cross-linked materials spectra suggests hydrogen bonds formation [30]. The analysis of bonds creation between aldehyde groups of DAS and amine groups of proteins is impossible because C=O stretching vibration band overlaps with Amide I.

**Table 1:** The position of main bands (cm^−1^) in FTIR spectra of collagen/elastin materials

Sample	Amide A	Amide B	CH_3_	Amide I	Amide II
95Coll-5El	3298	3083	2928	1631	1549
95Coll-5El-5DAS	3299	3082	2928	1631	1549
95Coll-5El-10DAS	3299	3077	2930	1632	1549
90Coll-10El	3300	3081	2928	1632	1549
90Coll-10El-5DAS	3299	3077	2926	1633	1549
90Coll-10El-10DAS	3300	3075	2926	1631	1549

### Thermal analysis

**Fig. 3:**
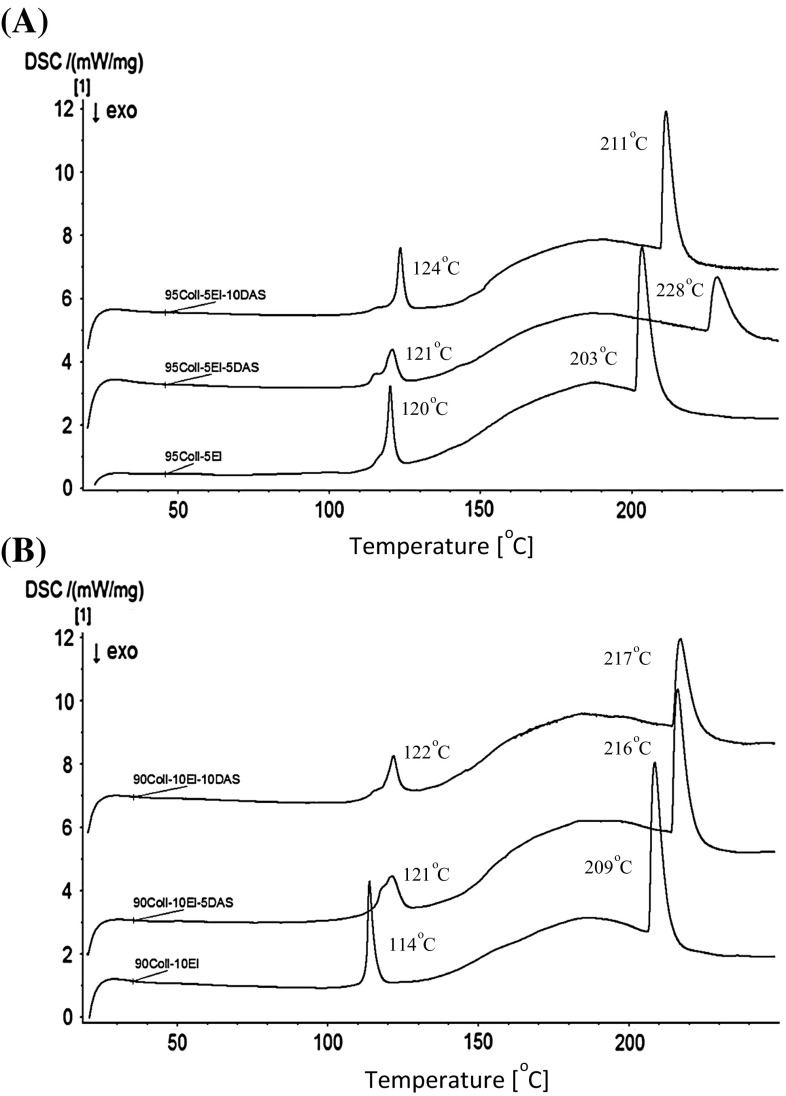
DSC curve of **a** 95Coll-5El, **b** 90Coll-10El materials

The thermal stability of collagen/elastin materials, unmodified and cross-linked by DAS, was tested with differential scanning calorimetry (DSC). As can be seen in Fig. 3, two endothermic peaks are observed during heating to 250 °C. The first one corresponds to the slow release of water associated with proteins. It is noticed at 120 °C for 95Coll-5El and at slightly lower temperature, 114 °C, in the case of 90Coll-10El. The temperature increases after cross-linking. It is then similar for the both kinds of samples. The second peak at around 203–228 °C results from the transformation of the ordered structure of the dry collagen/elastin materials into a random coil. It is induced by the thermal disruption of hydrogen and covalent bonds as well as other interactions inside the proteins structure. These temperatures are also higher for the samples containing DAS, which proves the occurrence of the cross-linking process [35–38].

### Mechanical properties

The mechanical properties of the collagen/elastin gels are also affected by the cross-linking process. The compression modulus for the unmodified samples containing 5 and 10 % of elastin is 377 and 359 kPa, respectively (Table 2). It shows that the higher addition of elastin hydrolysates destabilizes the collagen structure. As it was expected, cross-linking with DAS causes an increase in the compression modulus. However, it is worth noticing that higher changes are observed for the 90Coll-10El series, and consequently, the 90Coll-10El-10DAS gel is more rigid than 95Coll-5El-10DAS.

**Table 2:** The values of compression modulus of collagen/elastin materials cross-linked with dialdehyde starch

Sample	E (kPa)
95Coll-5El	3.77 ± 0.44
95Coll-5El-5DAS	3.56 ± 0.70
95Coll-5El-10DAS	4.63 ± 0.38^a^
90Coll-10El	3.59 ± 0.82
90Coll-10El-5DAS	4.85 ± 0.81^a^
90Coll-10El-10DAS	5.06 ± 0.58^a^

^a^Significantly different from non-cross-linked sample

### SEM

**Fig. 4:**
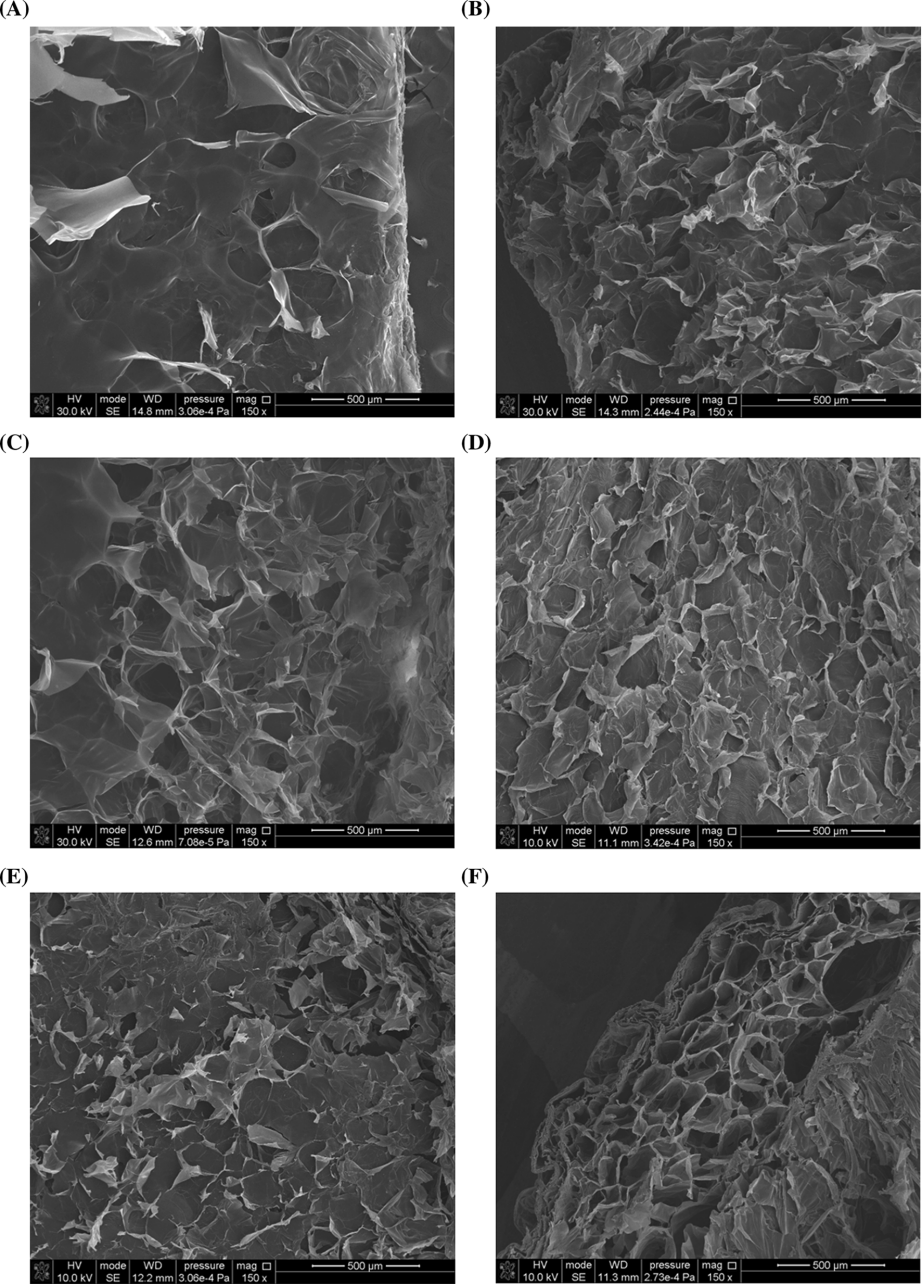
SEM images of lyophilized collagen/elastin gel. **a** 95Coll-5El, **b** 95Coll-5El-5DAS, **c** 95Coll-5El-10DAS, **d** 90Coll-10El, **e** 90Coll-10El-5DAS, **f** 90Coll-10El-10DAS

Porosity is one of the most important features of materials applied in tissue engineering. Hence, the SEM images of lyophilized collagen/elastin hydrogels, unmodified and cross-linked with DAS, were analysed (Fig. 4). The material possesses the 3-D porous structure. The pores have a rounded shape and are arranged longitudinally to the axis of the sample determined by the shape of the dialysis tube. The average size of the pores in the material is varied and depends on proteins composition and DAS addition (Table 3). The higher content of elastin results in the smaller pores area. Also, the cross-linking process causes the decrease in the pore size, proportionally to the amount of DAS used.

**Table 3:** The pore size of collagen/elastin materials cross-linked with dialdehyde starch

Sample	Pore size (µm)
95Coll-5El	444.46 ± 13.23
95Coll-5El-5DAS	401.32 ± 12.67
95Coll-5El-10DAS	375.55 ± 16.37
90Coll-10El	314.85 ± 13.49
90Coll-10El-5DAS	294.74 ± 7.11
90Coll-10El-10DAS	263.17 ± 13.53

### Swelling ability

**Fig. 5:**
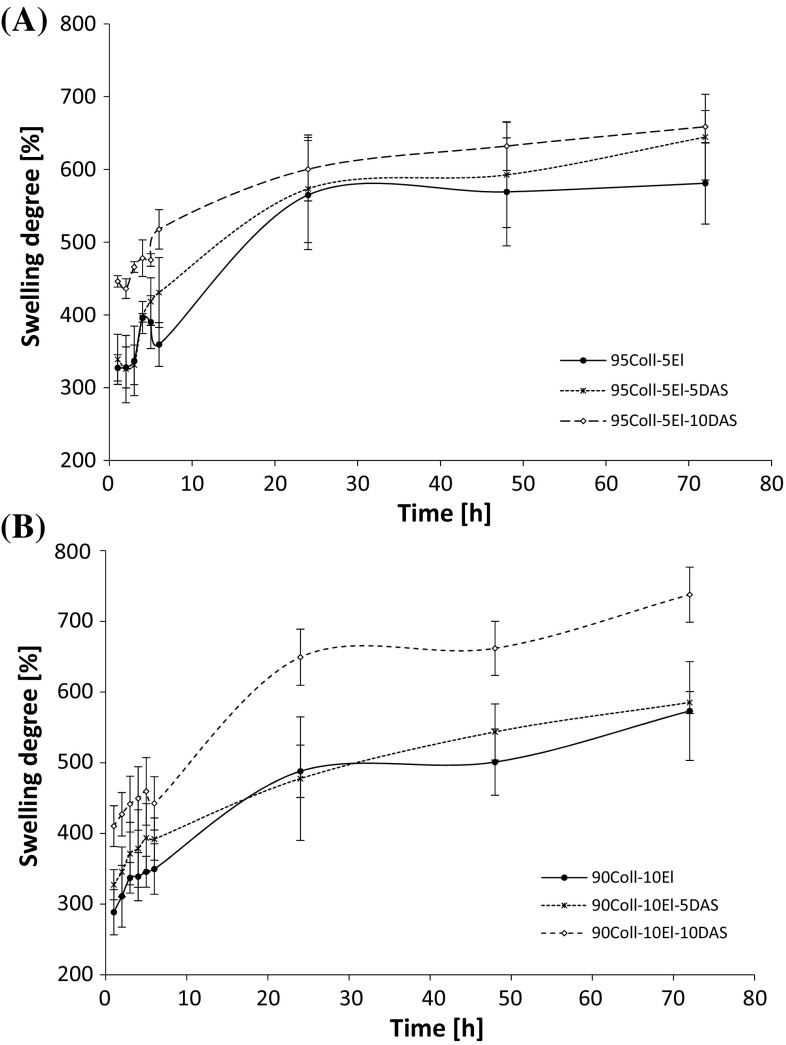
The swelling ratio E_s_ (%) of collagen/elastin gels. **a** 95Coll-5El, **b** 90Coll-10El

The collagen/elastin materials, due to the presence of a large number of functional groups, are easily wettable by polar solvents and exhibit higher swelling ability. During just 1 h of incubation, the gels absorb around 300–450 % of PBS, and after 24 h, the liquid content is stabilized (Fig. 5). The collagen/elastin materials cross-linking with DAS increases the swelling degree. The difference is higher for 10 % DAS addition, especially in the case of 90Coll-10El-10DAS.

### Surface properties

The character of a biomaterial surface is crucial for tissue-implant interactions. The adhesion of cells to the surface depends on various factors, e.g. the SFE and hydrophilicity. The contact angle measurements were carried out and SFE (IFT) as well as its polar and dispersive components were calculated with Owens–Wendt method (Table 4). The values of SFE are similar for all the samples and are in the range from 32 to 36 mJ/m^2^. Moreover, the polarity of the surfaces only slightly increase in the presence of DAS in the collagen/elastin gels.

**Table 4:** The surface free energy IFT(S) and its dispersive IFT (S,D) and polar component IFT(S,P) of collagen/elastin materials cross-linked with dialdehyde starch (calculated by Owens–Wendt method)

Sample	IFT(S)	IFT(S,D)	IFT(S,P)
95Coll-5El	36.0	25.57	10.47
95Coll-5El-5DAS	32.6	21.02	11.59
95Coll-5El-10DAS	34.7	25.21	9.48
90Coll-10El	33.4	24.28	9.15
90Coll-10El-5DAS	34.3	23.92	10.39
90Coll-10El-10DAS	35.0	22.69	12.26

### Enzymatic degradation

The life time of a material in the body depends on its enzymatic resistance. The collagen materials are resistant to the activity of many nonspecific enzymes. However, they may be degraded by collagenase, which cuts the peptide bonds between a neutral amino acid and glycine. This is a probable reason of a higher degradation process effectiveness in the case of 95Coll-5El gel when compared to 90Coll-10El (Table 5). Cross-linking significantly influences the rate of the protein materials degradation. Surprisingly, the addition of 5 % of DAS slightly increases the degradation ability in the both types of materials, and only the gels containing 10 % of DAS are more resistant. However, only the differences observed for 90Coll-10El are statistically significant.

**Table 5:** Enzymatic degradation of collagen/elastin materials cross-linked with dialdehyde starch

Sample	Degradation degree (%)
95Coll-5El	74.95 ± 1.78
95Coll-5El-5DAS	81.93 ± 4.88
95Coll-5El-10DAS	68.98 ± 4.80
90Coll-10El	66.74 ± 3.84
90Coll-10El-5DAS	87.25 ± 5.09^a^
90Coll-10El-10DAS	50.71 ± 3.52^a^

^a^Significantly different from non-cross-linked sample

### In vitro test

**Fig. 6:**
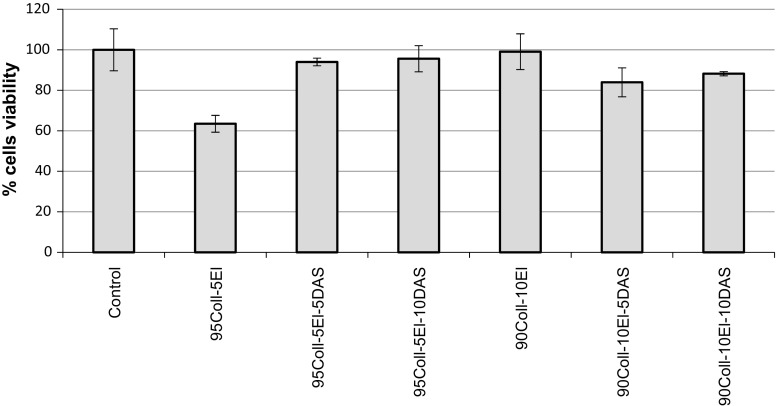
The in vitro test of 3T3 cells viability on collagen/elastin hydrogels before and after cross-linking with dialdehyde starch

In order to assess the biocompatibility of collagen/elastin hydrogels, the response of fibroblasts 3T3 cells growth was tested. After 7 days of incubation, the cells were well spread on the surface of the hydrogels. The fibroblasts viability was estimated by the qualitative analysis of colour intensity resulting from MTT assay. As one can see in Fig. 6, all the cross-linked materials are well tolerated by 3T3 cells. Moreover, DAS addition to 95Coll-5El improves the fibroblasts affinity and proliferation on the material.

## Discussion

**Fig. 7:**
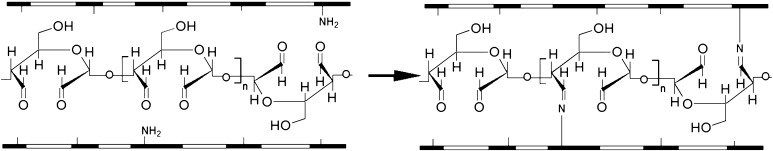
The formation of cross-linking bonds between aldehyde groups of DAS and amine groups of proteins

The presented method, combining two types of cross-linking techniques, allows obtaining the stable, stiff, transparent hydrogels. The dialysis of collagen/elastin solution against deionized water causes slow neutralization of the mixture. When the solution pH is about 7, it promotes the ionic interactions and hydrogen bonds formation between –NH and –C=O of peptide bonds and other groups of atoms present along the protein chains. Thus, the more ordered structure is created [28]. At the same time, the covalent cross-linking bonds between aldehyde groups of DAS and amine groups of proteins are formed (Fig. 7). The position of Amide I band at 1631 cm^−1^ in FTIR spectra is typical of the native collagen from rat tail tendons. This indicates that the neutralization process contributes to the formation of the triple helical structure [28, 34]. The addition of DAS causes no shift of the Amide I position. Additionally, the shape of the band remains the same. This demonstrates that the DAS reacts with amino acid residues outside of the triple helix and creates intermolecular bonds. It stays in accordance with the literature data stating that both low molecular and polymeric dialdehydes do not alter the secondary structure of collagen/elastin materials [20, 22].

In contrast to other authors, we did not observe the formation of more organized DAS structures such as fibrils when the collagen solution pH increased [39–43]. Instead, a kind of plate-like structure is created (SEM images). This probably results from a very slow neutralization rate (7 days) and gradual decrease in the ionic strength of the solution during dialysis process. Usually, in contrast to our method, the neutralization process is fast due to the use of alkaline reagents, and fibril formation is observed [39, 41, 42, 44]. This does not change the fact that all the tested materials show a porous structure on SEM images and the size of pores decrease after cross-linking with DAS.

Besides the microstructure, other properties of the hydrogels are also affected by the creation of covalent intermolecular cross-linking bonds. The DSC measurements show that the thermal stability of the materials increases due to DAS addition, as it was previously shown for collagen materials by Liu et al., Mu et al., and collagen hydrolysates by Langmaier [22, 24–26]. The temperature of water release and denaturation process is higher after cross-linking. The small differences between our data (temperature higher values) and other researchers can be noticed. This is caused by the fact that the thermal denaturation of proteins depends on a few factors, e.g. the number and organisation of inter- and intramolecular hydrogen bonds, water content, as well as cross-linking degree [37]. It is also worth noticing that the samples containing 10 % of elastin undergo bigger changes in comparison to the samples with lower elastin addition. This phenomenon is probably related to the fact, that the DAS reacts with amine groups of proteins. Elastin is richer in these groups because the hydrolysates chains are shorter, and it usually contains higher Lysine amount than collagen.

Some authors suggest that the higher thermal stability of collagen materials is related to its better biocompatibility and mechanical strength [26]. The results of our mechanical tests are consistent with this thesis. The cross-linked materials are more rigid and the increase in the compression modulus value is higher for 90Coll-10El samples rather than for 95Coll-5El. The presented data are close to the compression modulus obtained for neutralized collagen gels by Saddiq et al. and Achilli et al. [39, 40].

Interestingly, only the 10 % addition of DAS causes the decrease in the ability to enzymatic degradation, while the samples cross-linked with 5 % DAS are slightly less resistant. Surprisingly, the swelling ability slightly increases after collagen/elastin hydrogels cross-linking by DAS. Despite the fact that the more rigid structure is formed as DAS concentration is higher, a higher water absorption is observed. This is probably owing to the hydrophilic nature of DAS, which also swells easily in water. The slight increase in the surface polarity of the collagen/elastin materials containing 5 and 10 % of DAS confirms this suggestion. The calculated SFE values for all the tested materials are above 30 mJ/m^2^.

According to literature, such surfaces are considered suitable for tissue engineering. The satisfactory results of the in vitro test confirm this suggestion [45–50]. All the tested hydrogels are well tolerated by 3T3 cells in contrast to materials cross-linked by glutaraldehyde [1, 51, 52]. However, we have to bear in mind that the surface topography is also extremely important to cells affinity to biomaterial.

## Conclusion

The presented dual cross-linking method allowed us to obtain homogenous, transparent, relatively stiff hydrogels. The neutralization via dialysis process leads to the creation of a partially organized protein structure. However, owing to the slow rate of the process, the plate-like structure rather than fibrils is observed. The addition of 5 and 10 % of DAS to collagen/elastin mixtures causes the formation of intermolecular cross-linking bonds and does not affect the secondary structure of the proteins. As an effect, the thermal stability and mechanical strength of the modified hydrogels increase. However, only the materials containing 10 % of DAS are more resistant to enzymatic degradation. Moreover, the bigger changes for the 90Coll-10El than 95Coll-5El series are observed. This suggests that the effectiveness of cross-linking is higher for elastin than for collagen chains. The modification of the collagen/elastin hydrogels results in the higher swelling ability probably due to the polar nature of DAS. The values of the SFE and good affinity of 3T3 cells to the tested surfaces show that the materials are biocompatible. The presented results enable us to conclude that the DAS is an effective and safe cross-linking agent for collagen/elastin material. However, the high content of amine groups in protein is necessary. Thus, the cross-linking of elastin-rich materials (90Coll-10El) by DAS is more efficient.

## References

[1] Parenteau-Bareil R, Gauvin R, Berthod F (2010). Collagen-based biomaterials for tissue engineering applications. Materials.

[2] Chattopadhyay S, Raines RT (2014). Review collagen-based biomaterials for wound healing. Biopolymers.

[3] Dombi GW, Purohit K, Martin LM, Yang SC (2015). Collagen gel formation in the presence of a carbon nanobrush. J Mater Sci Mater Med.

[4] Sionkowska A, Kozlowska J (2013). Properties and modification of porous 3-D collagen/hydroxyapatite composites. Int J Biol Macromol.

[5] Daamen WF, Veerkamp JH, van Hest JCM, van Kuppevelt TH (2007). Elastin as a biomaterial for tissue engineering. Biomaterials.

[6] Daamen WF, van Moerkerk HT, Hafmans T, Buttafoco L, Poot AA, Veerkamp JH, van Kuppevelt TH (2003). Preparation and evaluation of molecularly-defined collagen-elastin-glycosaminoglycan scaffolds for tissue engineering. Biomaterials.

[7] Jarman-Smith ML, Bodymyali T, Stevens C, Howell JA, Horrocks M, Chaudhuri JB (2004). Porcine collagen crosslinking, degradation and its capability for fibroblast adhesion and proliferation. J Mater Sci Mater Med.

[8] He L, Mu C, Shi J, Zhang Q, Shi B, Lin W (2011). Modification of collagen with natural cross-linker, procyanidin. Int J Biol Macromol.

[9] Vaz CM, De Graaf LA, Reis RL, Cunha AM (2003). Effect of crosslinking, thermal treatment and UV irradiation on the mechanical properties and in vitro degradation behavior of several natural proteins aimed to e used in the biomedical field. J Mater Sci Mater Med.

[10] Suh H, Park JC (2000). Evaluation of calcification in porcine valves treated by ultraviolet ray and glutaraldehyde. Mater Sci Eng C.

[11] Chan BP, Chan OC, So KF (2008). Effects of photochemical stability on the microstructure of collagen and a feasibility study on controlled protein release. Acta Biomater.

[12] Rich H, Odlyha M, Cheema U, Mudera V, Bozec L (2014). Effect of photochemical riboflavin- mediated crosslinks on the physical properties of collagen constructs and fibrils. J Mater Sci Mater Med.

[13] Boekema BK, Vlig M, OldeDamnik L, Middelkoop E, Eummelen L, Buhren AV, Ulrich MM (2005). Effect of pore size and cross-linking of a novel collagen-elastin dermal substitute on wound healing. J Mater Sci: Mater Med..

[14] Chen RN, Ho HO, Sheu MT (2005). Characterization of collagen matrices crosslinked using microbial transglutaminase. Biomaterials.

[15] Wong SS, Jameson DM (2012). Chemistry of protein and nucleic acid cross-linking and conjugation.

[16] Sereikaite J, Bassus D, Bobnis R, Dienys G, Bumeliene Z, Bumelis VA (2003). Divinyl sulfone as a crosslinking reagent for oligomeric proteins. Bioorg Khim.

[17] Heijmen FH, du Pont JS, Middelkoop E, Kreis RW, Hoekstra MJ (1997). Cross-linking of dermal sheep collagen with tannic acid. Biomaterials.

[18] Kozlowska J, Sionkowska A (2015). Effects of different crosslinking methods on the properties of collagen-calcium phosphate composite materials. Int J Biol Macromol.

[19] Olde Damnik LHH, Dijkstra PJ, Van Luyn MJA, Van Wachem PB, Nieuwenhuis P, Fejen J (1995). Glutaraldehyde as crosslinking agent for collagen-based biomaterials. J Mater Sci: Mater Med..

[20] Fathima NN, Madhan B, Rao JR, Nair BU, Ramasami T (2004). Interaction of aldehydes with collagen: effect on thermal, enzymatic and conformational stability. Int J Biol Macromol.

[21] Fathima NN, Baias M, Blumich B, Ramasami T (2010). Structure and dynamics of water in native and tanned collagen fibers: effect of crosslinking. Int J Biol Macromol.

[22] Mu C, Liu F, Cheng Q, Li H, Wu B, Zhang G, Lin W (2010). Collagen cryogel cross-linked by dialdehyde starch. Macromol Mater Eng.

[23] Song L, Cruz C, Farrah SR, Baney RH (2009). Novel antiviral activity of dialdehyde starch. Electron J Biotechnol.

[24] Langmaier F, Mladek M, Mokrejs P, Kolomaznik K (2008). Biodegradable packing materials based on waste collagen hydrolysate cured with dialdehyde starch. J Therm Anal Calorim.

[25] Langmaier F, Mladek M, Mokrejs P (2008). Hydrogels of collagen hydrolysate cross-linked with dialdehyde starch. J Therm Anal Calorim.

[26] Liu Y, Acharya G, Lee CH (2011). Effects of dialdehyde starch on calcification of collagen matrix. J Biomed Mater Res A..

[27] Sionkowska A, Skopinska-Wisniewska J, Gawron M, Kozlowska J, Planecka A (2010). Chemical and thermal cross-linking of collagen and elastin hydrolysates. Int J Biol Macromol.

[28] Skopinska-Wisniewska J, Olszewski K, Bajek A, Rynkiewicz A, Sionkowska A (2014). Dialysis as a method of obtaining neutral collagen gels. Mater Sci Eng C.

[29] Zenkiewicz M (2007). Methods for the calculation of surface free energy of solids. J Achiev Mater Manuf Eng.

[30] Stuart BH (2004). Infrared spectroscopy: fundamentals and applications.

[31] Kong J, Yu S (2007). Fourier transform infrared spectroscopic analysis of protein secondary structures. Acta Biochim Biophys Sin.

[32] Barth A (2007). Infrared spectroscopy of proteins. Biochim Biophys Acta.

[33] Cucos A, Budrugeac P, Mitrea S (2013). The influence of sodium chloride on the melting temperature of collagen crystalline region in parchments. J Therm Anal Calorim.

[34] de Campos Vidal B, Mello MLS (2011). Collagen type I amide I band infrared spectroscopy. Micron.

[35] Cucos A, Budrugeac P (2014). Simultaneous TG/DTG-DSC-FTIR characterization of collagen in inert and oxidative atmospheres. J Therm Anal Calorim.

[36] Safandowska M, Pietrucha K (2013). Effect of fish collagen modification on its thermal and rheological properties. Int J Biol Macromol.

[37] Usha R, Ramasami T (2004). The effects of urea and n-propanol on collagen denaturation: using DSC, circular dichroism and viscosity. Thermochim Acta.

[38] Miles CA, Avery NC, Rodin VV, Bailey AJ (2005). The increase in denaturation temperature following cross-linking collagen is caused by dehydration of the fibres. J Mol Biol.

[39] Achilli M, Mantovani D (2010). Tailoring mechanical properties of collagen-based scaffolds for vascular tissue engineering: the effects of pH, temperature and ionic strength on gelation. Polymers.

[40] Saddiq ZA, Barbenel JC, Grant MH (2005). The mechanical strength of collagen gels containing glycosaminoglycans and populated with fibroblasts. Eur Cells Mater..

[41] Harris JR, Reiber A (2007). Influence of saline and pH on collagen type I fibrillogenesis in vitro: fibril polymorphism and colloidal gold labelling. Micron..

[42] Gobeaux F, Mosser G, Anglo A, Panine P, Davidson P, Giraud-Guille MM, Belamie E (2008). Fibrillogenesis in dense collagen solutions: a physicochemical study. J Mol Biol.

[43] Raub CB, Unruh J, Suresh V, Krasieva T, Lindmo T, Gratton E, Tromberg BJ, George SC (2008). Image correlation spectroscopy of multiphoto images correlates with collagen mechanical properties. Biophys J.

[44] Saito H, Murabayashi S, Mitamura Y, Tageuchi T (2008). Characterization of alkali-treated collagen gels prepared by different crosslinkers. J Mate Sci: Mater Med..

[45] Liber-Knec A, Lagan S (2014). The use of contact angle and the surface free energy as the surface characteristics of the polymers used in medicine. Polym Med.

[46] Xu LC, Siedlecki CA (2007). Effects of surface wettability and contact time on protein adhesion to biomaterial surfaces. Biomaterials.

[47] Gentleman MM, Gentleman E (2014). The role of surface free energy in osteoblast–biomaterial interactions. Int Mater Rev.

[48] Parhi P, Golas A, Vogler EA (2010). Role of proteins and water in the initial attachment of mammalian cells to biomedical surfaces: a review. J Adhes Sci Technol.

[49] Menzies KL, Jones L (2010). The impact of contact angle on the biocompatibility of biomaterials. Optom Vis Sci.

[50] Skopinska-Wisniewska J, Sionkowska A, Kaminska A, Kaznica A, Joachimiak R, Drewa T (2009). Surface characterization of collagen/elastin based biomaterials for tissue regeneration. Appl Surf Sci.

[51] Sheu MT, Huang JC, Yeh GC, Ho HO (2001). Characterization of collagen gel solutions and collagen matrices for cell culture. Biomaterials.

[52] Gough JE, Scotchford CA, Downes S (2002). Cytotoxicity of glutaraldehyde crosslinked collagen/poly(vinyl alcohol) films is by the mechanism of apoptosis. J Biomed Mater Res.

